# Identification of the B-Raf/Mek/Erk MAP kinase pathway as a target for all-trans retinoic acid during skin cancer promotion

**DOI:** 10.1186/1476-4598-8-27

**Published:** 2009-05-11

**Authors:** Satish B Cheepala, Weihong Yin, Zanobia Syed, Jennifer N Gill, Alaina McMillian, Heather E Kleiner, Mark Lynch, Rasiah Loganantharaj, Marjan Trutschl, Urska Cvek, John L Clifford

**Affiliations:** 1 Department of Biochemistry, Louisiana State University Health Sciences Center-Shreveport and Feist-Weiller Cancer Center, 1501 Kings Hwy, Shreveport, Louisiana, 17730, USA; 2 St Jude Children's Research Hospital, Memphis, TN 38105, USA; 3 Wake Forest University School of Medicine, Winston-Salem, NC 27157, USA; 4 Pharmacology, Louisiana State University Health Sciences Center-Shreveport and Feist-Weiller Cancer Center, 1501 Kings Hwy, Shreveport, Louisiana, 17730, USA; 5 Department of Cancer Biology, Bayer HealthCare Pharmaceuticals, West Haven, Connecticut, USA; 6 Bioinformatics Research Lab, University of Louisiana at Lafeyette, PO Box 44330, Lafayette, LA 70504, USA; 7 Department of Computer Science, Louisiana State University-Shreveport, USA

## Abstract

**Background:**

Retinoids have been studied extensively for their potential as therapeutic and chemopreventive agents for a variety of cancers, including nonmelanoma skin cancer (NMSC). Despite their use for many years, the mechanism of action of retinoids in the prevention of NMSC is still unclear. In this study we have attempted to understand the chemopreventive mechanism of all-*trans *retinoic acid (ATRA), a primary biologically active retinoid, in order to more efficiently utilize retinoids in the clinic.

**Results:**

We have used the 2-stage dimethylbenzanthracene (DMBA)/12-*O*-tetradecanoylphorbol-13-acetate (TPA) mouse skin carcinogenesis model to investigate the chemopreventive effects of ATRA. We have compared the gene expression profiles of control skin to skin subjected to the 2-stage protocol, with or without ATRA, using Affymetrix 430 2.0 DNA microarrays. Approximately 49% of the genes showing altered expression with TPA treatment are conversely affected when ATRA is co-administered. The activity of these genes, which we refer to as 'counter-regulated', may contribute to chemoprevention by ATRA. The counter-regulated genes have been clustered into functional categories and bioinformatic analysis has identified the B-Raf/Mek/Erk branch of the MAP kinase pathway as one containing several genes whose upregulation by TPA is blocked by ATRA. We also show that ATRA blocks signaling through this pathway, as revealed by immunohistochemistry and Western blotting. Finally, we found that blocking the B-Raf/Mek/Erk pathway with a pharmacological inhibitor, Sorafenib (BAY43-9006), induces squamous differentiation of existing skin SCCs formed in the 2-stage model.

**Conclusion:**

These results indicate that ATRA targets the B-Raf/Mek/Erk signaling pathway in the 2-stage mouse skin carcinogenesis model and this activity coincides with its chemopreventive action. This demonstrates the potential for targeting the B-Raf/Mek/Erk pathway for chemoprevention and therapy of skin SCC in humans. In addition our DNA microarray results provide the first expression signature for the chemopreventive effect of ATRA in a mouse skin cancer model. This is a potential source for novel targets for ATRA and other chemopreventive and therapeutic agents that can eventually be tested in the clinic.

## Background

Nonmelanoma skin cancer (NMSC) is the most common cancer in the U.S., with over a million new cases of the two most common forms, squamous cell carcinoma (SCC) and basal cell carcinoma (BCC), anticipated annually [1]. The more clinically aggressive form is SCC [2], which has been increasing in incidence since the 1960s at annual rates from 4% to as much as 10% in recent years. Advanced-disease- and treatment-related morbidity have a profound impact on patients' quality of life. Unlike BCC, which bears a single genetic hallmark of disruption of the patched-sonic hedgehog signaling pathway, the genetic alterations leading to SCC appear more complex and varied, and are poorly understood [3]. Better control of advanced skin SCC is clearly necessary, and will be greatly helped by improving our understanding of the molecular basis for skin carcinogenesis and of the action of chemopreventive drugs.

The mouse skin model of multi-stage carcinogenesis is one of the best studied and most informative with regard to understanding molecular mechanisms and the evolution of cancer cells [4]. It has proven to be ideal for the study of events leading to the transition from initiation, to promotion and then progression to carcinoma. Molecular analysis of multistage human cancers such as prostate and colon cancer, have shown a high level of genetic and biological similarity to mouse skin in the 2-stage model. [5]. The SENCAR (sensitive to carcinogen) mouse strain has been developed for this assay due to its high susceptibility to chemical-induced tumor formation, relative to most other strains of mice tested [4].

Retinoids comprise a class of chemical compounds that includes active metabolites of vitamin A (retinol), as well as a diverse array of synthetic derivatives. Retinoids modulate a several cellular processes, including proliferation, differentiation, homeostasis, malignant transformation and apoptosis [6]. Retinoids also act pharmacologically to restore regulation of differentiation and growth in certain premalignant and malignant cells *in-vitro *and *in-vivo*. Consequently, retinoids are being studied extensively for their potential as therapeutic and chemopreventive agents for a variety of cancers including skin SCC [7].

It has been previously shown that all-trans retinoic acid (ATRA), the primary biologically active retinoid found in the body, reduced both the number of papillomas and carcinomas that formed in the 2-stage model [8,9]. A possible mechanism for this effect is revealed from studies of the AP-1 transcription factor complex, which is comprised of heterodimers of jun and fos protein family members. AP-1 is a positive regulator of cell proliferation and transformation and its activity is stimulated by 12-*O*-tetradecanoylphorbol-13-acetate (TPA) [10]. The promotion step is likely to involve AP-1 since the oncogenic forms of c-fos and p21^ras ^have been shown to cooperate in converting normal keratinocytes to squamous cell carcinomas [11]. The requirement for AP-1 activation for tumor promotion was subsequently shown using transgenic mice expressing the *c-jun *transactivation mutant TAM67, under the control of the basal cell specific keratin 14 promoter [12]. As would be predicted from the antagonism between TPA and retinoids, the nuclear retinoid receptors and AP-1 can antagonize each other's activity for the regulation of several genes [13,14]. It has further been shown that the tumor suppressive effect of ATRA in the 2-stage model is mediated by blocking AP-1 activity, and not through transcriptional activation of retinoic acid responsive genes *per se *[14,15]. Finally, a direct link between between MAPK signaling and AP-1 activity has been established from studies in which kinase deficient forms of Erk could inhibit AP-1 activation by several stimuli [16,17]. These findings have led to several studies aimed at understanding the mechanism of suppression of MAP kinase signaling and/or AP-1 activity by ATRA [18].

The development of DNA microarray technology provides a powerful tool for identifying genes expressed in a given cell type or tissue at a particular time [19]. In this study we have used this technology to not only determine the effect of a tumor promoting agent (TPA) on normal tissue, but also to assess the effects of ATRA, a known suppressor of TPA-induced tumor promotion, in this setting. We have identified a set of genes that we have termed 'counter-regulated' by TPA and ATRA. These are TPA regulated genes (fold expression change ≥ |1.5| between control and TPA treated skin), which are expressed in the opposite direction when TPA is co-administered with ATRA (fold change ≥ |1.5| between skin treated with TPA+ATRA and TPA alone). Such genes provide an expression signature for the chemopreventive effect of ATRA in the mouse 2-stage skin cancer model. A prominent component of this signature is the B-Raf/Mek/Erk branch of the MAP kinase signaling pathway.

## Results

### ATRA reverses many of the gene expression changes caused by TPA in mouse skin: microarray-based analysis

Our group and others have shown that ATRA can suppress tumor promotion by TPA in the 2-stage model, but only as long as it is being co-administered with TPA [9,20]. If ATRA treatment is discontinued while TPA treatment is maintained, the mice showed a linear increase in tumor multiplicity with time after stopping the last treatment [20]. This suggests that ATRA may be primarily acting to suppress promotion by TPA, but has little apparent effect on initiation.

**Figure 1:**
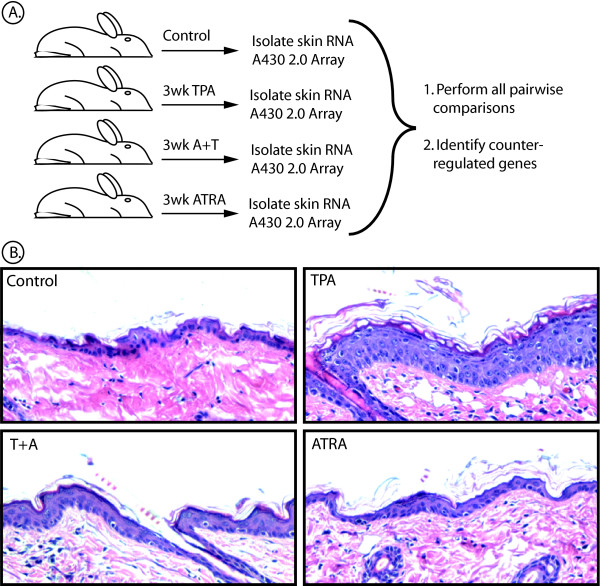
**Experimental design of microarray analysis**. *A*, Mouse skin was treated twice weekly for 3 weeks with either 200 μl acetone solvent (Control), 1 μg TPA in 200 μl acetone (TPA), 5 μg ATRA in 200 μl acetone (ATRA) or ATRA administration followed immediately by TPA (T+A). *B*, Hematoxylin and eosin stain of adjacent skin samples from mice in the same treatment groups used for RNA isolation.

In order to explore the mechanism of the chemopreventive effect of ATRA at an early step during TPA-induced tumor promotion, we performed Affymetrix DNA microarray analyses for mouse skin subjected to the 2-stage protocol for 3 weeks, with and without co-administration of ATRA. We chose the 3 week time point because this precedes the appearance of tumors but is after the TPA treated skin exhibits pronounced hyperplasia (Fig. 1); and this time point proved to be advantageous for identifying genes regulated by TPA plus retinoid combinations in mouse skin in a previous study from our laboratory [20]. We reason that at least a portion of important TPA-induced promotion events will have taken place by this time.

**Figure 2:**
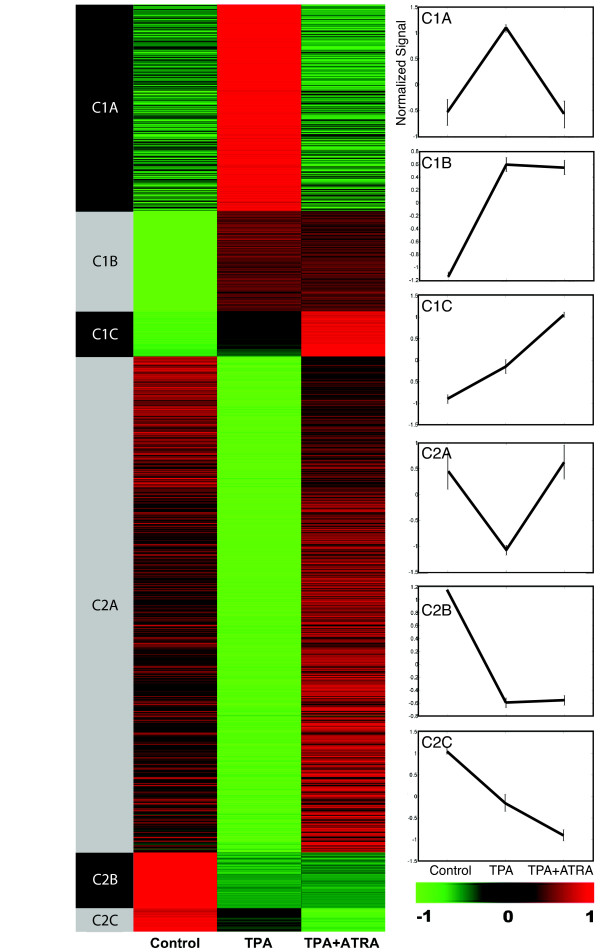
**Heatmap of identified clusters**. SENCAR mouse skin was treated with TPA or TPA + ATRA. Expression of genes induced ≥ 1.5-fold by TPA treatment compared to controls (C1A-C subclusters) and suppressed ≥ 1.5 fold by TPA (C2A-C) are indicated in green-red scale (green = low expression, red = high expression, black = intermediate expression). Expression patterns of genes for each subgroup are shown in graphs on the right, in which the raw scores were normalized to a mean value of 0 and a standard deviation of 1.

Of the ~45,000 probe sets (representing ~39,000 transcripts and variants from over 34,000 characterized mouse genes) on the 430 2.0 GeneChip, expression of 3,948 were altered by TPA, upwards or downwards, relative to untreated controls (fold changes between treatment groups ≥ 1.5, with p ≤ 0.05). This group of genes was divided into clusters as described in Materials and Methods. The heatmap in figure 2 represents the genes that fall into each subcluster, with expression patterns produced by normalized raw scores for the subcluster indicated in the panels on the right. Amongst the 3,948 total TPA regulated genes, 49.5% were found to be in the C1A and C2A subclusters. We are calling these genes 'counter-regulated' because their direction of expression (increase or decrease) in TPA + ATRA treated skin compared to TPA treated skin is opposite to that of TPA treated skin compared to control skin. This data can also be represented graphically as scatterplots (Fig. S1, additional file 1). We note that when comparing control skin with TPA + ATRA skin, the majority of counter-regulated genes lie close the x = y diagonal, indicating that ATRA can suppress TPA effects on expression of these genes completely or near completely (Fig. S1C, additional file 1, see C1A and C2A subclusters). A subset of the C1A and C2A genes are listed in Table S1 in additional file 2, sorted according to their known function in cellular signaling pathways, using the GeneSifter program. The table lists genes in the major signaling pathways in epithelial cells that have the highest Z-scores. Z-score is a measure of the significance of over-representation for a particular gene list within an ontology group [21]. We also identified several counter-regulated genes that were previously determined to be TPA regulated (Table S2, additional file 3). This abrogation of TPA effect on gene expression by ATRA is consistent with the findings of numerous investigators who have shown that TPA activates the AP-1 transcription factor complex, and that AP-1 activity is inhibited by ATRA through the retinoid receptors [10] for review and [14,22].

### ATRA suppresses B-Raf/Mek/Erk signaling in early and late tumor progression times in the 2-stage skin carcinogenesis model

**Figure 3:**
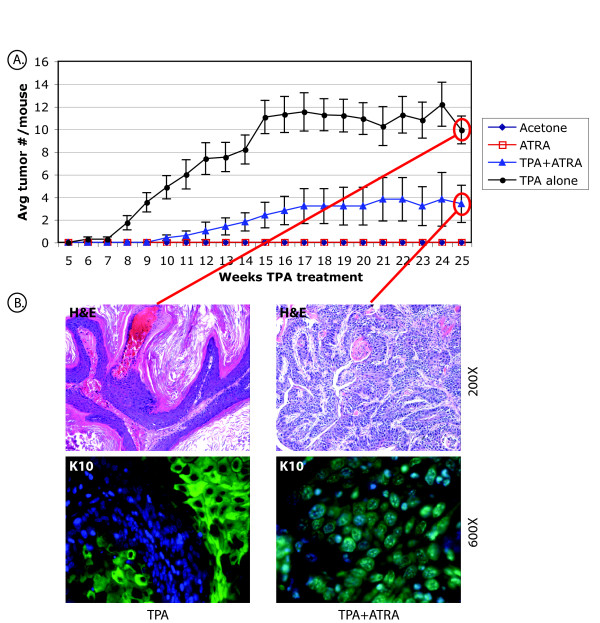
**Chemopreventive activity of ATRA in the 2-stage model**. *A*, Tumor multiplicity for mice in the 2-stage skin carcinogenesis experiment. Tumors were counted weekly. Error bars represent +/- SEM for each treatment group (n = 5). *B*, H&E stain of paraffin sections from representative tumors isolated from mice in the TPA alone and TPA+ATRA 30 week treatment groups. Magnification = 200×. *C*, IHC staining of serial sections from the same tumors with an antibody to cytokeratin 10 followed by an Alexa-488-labeled secondary antibody and counterstaining with DAPI. Magnification = 600×.

**Figure 4:**
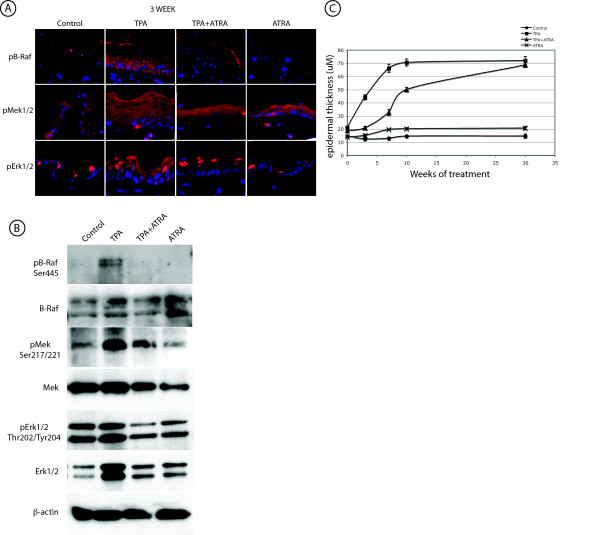
**ATRA suppresses TPA induced phosphorylation of B-Raf, Mek1/2, and Erk1/2**. *A*. IHC staining of 3 week treated skin. Paraffin sections of mouse skin treated with Acetone (Control), TPA, TPA+ATRA, and ATRA alone were probed with pB-Raf, pMek1/2, and pERK1/2 antibodies followed by an Alexa-546-labeled secondary antibody. Sections were counterstaining with DAPI. All panels were photographed at 600× magnification. *B*. Epidermal lysates were prepared in RIPA buffer from SENCAR mouse skin treated with Acetone (Con), TPA, TPA+ATRA (T+A), or ATRA alone for 10 weeks. Total protein (15 μg per well) from pooled samples (n = 5) was run on 10% SDS-PAGE and probed with antibodies for total B-Raf, pB-Raf, total Mek1/2, pMek1/2, total Erk1/2, pERK1/2, and β-actin. *C*. Epidermal thickness during the time course. Thickness was measured from digital micrographs of H&E stained skin sections as described in Methods. The symbols and error bars indicate the mean of 10 measurements ± SEM.

Examination of the above microarray results revealed altered expression of a disproportionate number of genes involved in the MAP kinase signaling pathway, in particular the B-Raf/Mek/Erk pathway (Table S1, additional file 2). We next performed a 2-stage skin carcinogenesis experiment as above, except that in this study the protocol was carried out for 30 weeks. Tumor counts were recorded each week and mice were sacrificed at multiple time points (6 hours, 3, 7, 10, and 30 weeks of treatment), for subsequent analysis of B-Raf/Mek/Erk pathway signaling. The reduced tumor multiplicity and increased tumor latency for mice treated with TPA+ATRA, compared to mice treated with TPA alone, indicated the potent tumor suppressive activity of ATRA, in agreement with previous results (Fig. 3A, compare black line with blue line) [8,9]. However a small number of tumors were observed in the mice treated with TPA+ATRA. Interestingly, histological examination of these ATRA resistant tumors revealed a less differentiated phenotype than for the tumors that arose in the mice treated with TPA alone (Fig. 3B). These tumors closely resembled advanced SCC, while the tumors in the TPA mice were papillary, with a high degree of keratinization as confirmed by a immunohistochemical (IHC) staining for cytokeratin 10 (Fig. 3B, lower panels). In order to investigate the effects of ATRA on TPA-induced activation of the B-Raf/Mek/Erk pathway, we performed IHC staining of paraffin sections from mouse skin treated with TPA, ATRA, TPA+ATRA, or acetone solvent, with phosphorylation site-specific antibodies to B-Raf (phospho-Ser 445), Mek1/2 (phospho-Ser 217/221), and Erk1/2 (phospho-Thr202/Tyr 204). The 6 h, 3, 7, 10 and 30 week time points were selected for sample isolation in order to obtain data prior to tumor formation (6 h and 3 weeks), just as tumors are beginning to be detectable (7 weeks), after the majority of mice have tumors (10 weeks), and after tumors had grown substantially (30 weeks). In the TPA treated skin, hyperproliferation was correlated with increased levels of activated (phosphorylated) B-Raf, Mek1/2, and Erk1/2, as detected by increased red fluorescence from the Alexa 546-coupled secondary antibody. Results are shown for a representative (3 week) time point (Fig. 4A; compare TPA panels to Control panels for all 3 rows). Skin treated with a single application of TPA, ATRA or TPA+ATRA showed no notable changes in staining for any of the phosphoproteins (data not shown). pB-Raf was expressed predominantly in the cytoplasm in TPA treated skin at all time points, and this effect was blocked with co-administration of ATRA with TPA (Fig. 4A, upper rows and data not shown). We did not observe any changes in phosphorylation of c-Raf (RAF1), the other Raf family member present in skin, with any of the treatments using an antibody to phospho-serine 338 (Fig. S2, additional file 4).

pMek1/2 levels were increased and expression was localized predominantly to the inner surface of the cell membrane in TPA treated skin (Fig. 4A, middle rows). This observation is notable since it has been shown that the translocation of pMek to the cell membrane results in lowering the threshold for activation of MAP kinase signal output [23]. As was observed for pB-Raf, the increased levels of pMek1/2 was blocked with co-administration of ATRA. Staining for pErk1/2 was observed in pairs of scattered nuclei in control skin, with an increase in staining intensity for TPA treated skin, as would be predicted accompanying hyperproliferation. Fewer nuclei were stained for TPA+ATRA treated skin than for TPA treatment alone (Fig. 4A, bottom rows). We attempted to quantitate pErk1/2 levels by determining the percent positive nuclei in the treated skin. At all time points except 30 weeks, the percent positive pErk staining nuclei actually decreased in TPA treated skin compared to controls and TPA+ATRA skin (data not shown). This was due to the far greater number of total cells in the hyperplastic TPA treated skin.

To more precisely measure the levels of phosphorylation of the MAP kinase signaling proteins in this model, we performed Western blot analysis on epidermal lysates pooled from 5 mice per treatment group for each time point, from skin adjacent to the tissue that was embedded for sectioning and IHC. Using 10 weeks as a representative time point, co-administration of ATRA with TPA resulted in a lower level of pB-Raf, pMek1/2 and pErk1/2, than with TPA treatment alone (Fig. 4B compare TPA and TPA+ATRA lanes). At the same time there is an increase in the protein levels of total Erk1/2 with TPA treatment that is suppressed by ATRA. The results from both IHC and Western blot analysis indicate that suppression of pB-Raf, pMek1/2, and pErk1/2 levels by ATRA.

### P38 and JNK branches of the MAP Kinase cascade are not suppressed by ATRA treatment

**Figure 5:**
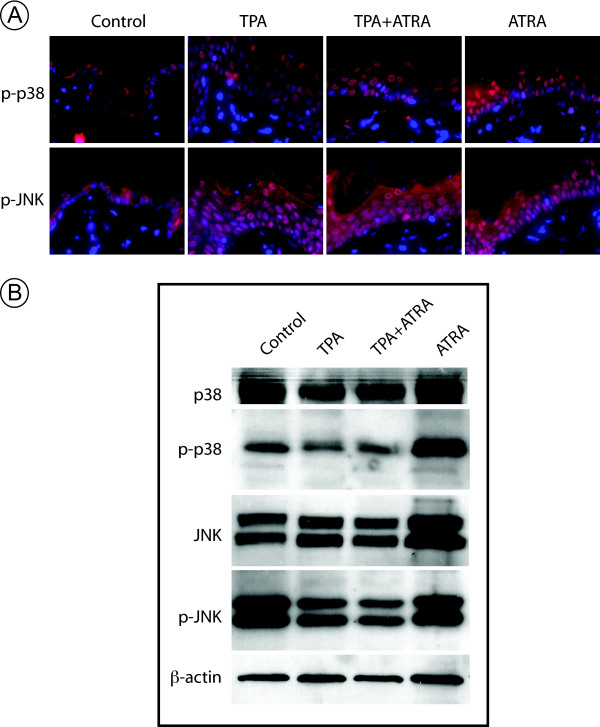
**TPA does not induce p38 and JNK phosphorylation**. *A*. Paraffin sections of SENCAR mouse skin treated for 7 weeks with Acetone (Control), TPA, TPA+ATRA, and ATRA alone were probed with phospho-p38 and phospho-JNK antibodies followed by an Alexa-546-labeled secondary antibody and DAPI counterstain. All panels in *A *were photographed at 600× magnification. *B*. Western blots were run as in Fig. 4E for 7 week epidermal lysates and probed with antibodies for total p38, phospho-p38 (p-p38), total JNK, phospho-JNK (p-JNK) and β-actin.

The two other major MAP kinase signaling pathways besides the B-Raf/Mek/Erk pathway are the Jun N-terminal kinase (JNK) and p38 MAP kinase pathways. In order to confirm our previous microarray results, which suggested that these pathways were less important in regards to ATRA suppression of TPA induced tumor promotion, we performed IHC on tissue sections taken at the 7 week time point from the same 2-stage skin carcinogenesis experiment as above. Using antibodies to phosphorylated (activated) p38 (Thr180/Tyr182), and JNK (Thr183/Tyr185) we observed that, unlike the B-Raf, Mek1/2 and Erk1/2 proteins, neither p38 nor JNK were increased in phosphorylation upon TPA treatment (Fig. 5A, compare TPA panels to Control). Importantly, the phosphorylation levels of p38 and JNK were not suppressed by co-administration of ATRA with TPA (Fig. 5A, compare TPA and TPA+ATRA panels). This observation was confirmed by Western blotting using the same phospho-specific antibodies, as well as antibodies to total p38 and total JNK (Fig. 5B). Interestingly we observed a modest increase in p38 phosphorylation in the skin treated with ATRA alone, suggesting that the p38 pathway could play a role in mediating retinoid effects in skin.

### Sorafenib suppresses cell growth in TPA-induced tumors

We hypothesize that ATRA exerts its tumor suppressive effect by blocking B-Raf/Mek/Erk signaling. This predicts that blocking this pathway through another means should also suppress tumorigenesis and/or the growth of tumors. To test this prediction we have used a novel Raf inhibiting bi-aryl urea compound from Bayer Pharmaceuticals called BAY 43-9006, or Sorafenib [24]. This compound has been shown to inhibit MAP kinase signaling in colon, pancreatic and breast cancer cell lines, as well as inhibit tumor growth in colon, breast and non-small-cell lung cancer mouse xenograft models.

**Figure 6:**
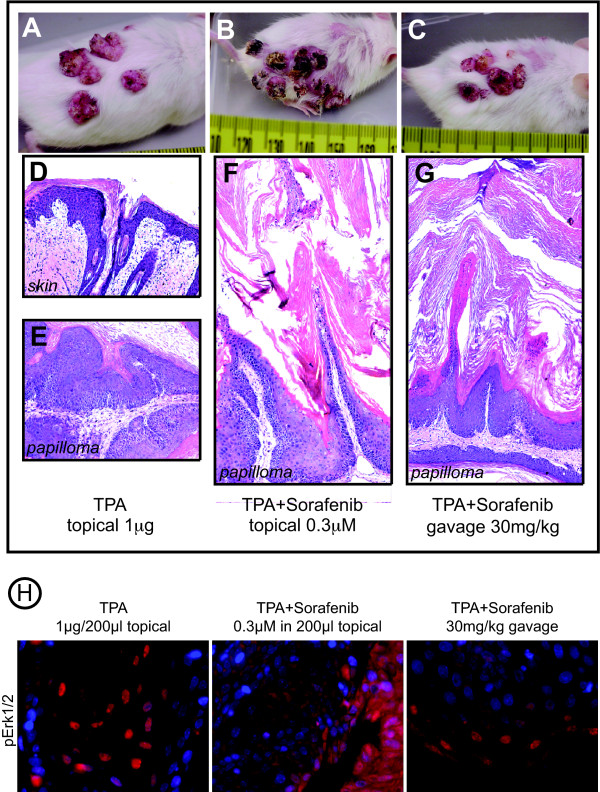
**Macroscopic appearance of papillomas from mice treated topically with TPA (1 μg/200 μl acetone, twice weekly) for 27 weeks, followed by an additional 3 weeks of TPA treatment alone (*A*), 3 weeks of TPA co-administered with 0.3 μM Sorafenib applied directly to the tumors (*B*) or TPA with 30 mg/kg Sorafenib administered by oral gavage (*C*)**. H&E staining of 30 week TPA treated skin (*D*), papillomas treated with TPA alone (*E*), TPA + topical Sorafenib (*F*), and TPA + oral gavage administered Sorafenib (*G*). *H*. IHC staining of papillomas from mice treated topically with TPA for 27 weeks, followed by an additional 3 weeks of TPA treatment alone (left panel), 3 weeks of TPA co-administered with topical 0.3 μM Sorafenib (middle panel) or TPA with 30 mg/kg Sorafenib administered by oral gavage (right panel). Sections were probed with an anti-pErk1/2 antibody followed by an Alexa-546 labeled secondary antibody, and counterstained with DAPI. Sections were photographed at 600× magnification.

Tumor-bearing SENCAR mice that had been treated topically with TPA, twice weekly for 27 weeks, were treated with Sorafenib either topically or by gavage, as described in the Materials and Methods. Mice were photographed twice weekly to monitor tumor morphology and size. Tumors that received only topical acetone solvent, along with the TPA treatment, displayed the exophytic papilloma morphology normally observed in TPA-treated mice in this model (Fig. 6A). The histological appearance of these tumors was also typical of TPA-induced papillomas (Fig. 6E). Tumors receiving topical Sorafenib at either concentration displayed a dried and darkened appearance that was reminiscent of human keratoacanthoma (Fig. 6, compare A to B and data not shown). Histological analysis of these tumors revealed that the bulk of the tumor mass was non-cellular keratin, indicating a greatly enhanced level of squamous differentiation compared to the control TPA-induced tumors (Fig. 6, compare E to F). Interestingly, the tumors of mice treated with Sorafenib by oral gavage did not differ drastically in outward appearance from the control TPA-induced tumors. However upon histological examination these tumors appear very similar to the tumors that received topical Sorafenib (Fig. 6C and 6G). It was previously shown in the breast and colon tumor xenograft models that inhibition of tumor growth was closely associated with suppression of Erk1/2 phosphorylation [24]. We therefore stained paraffin-embedded sections of the TPA-induced tumors, with and without Sorafenib treatment, with the pErk1/2 antibody. The number of nuclei and intensity of staining was reduced for Sorafenib treated tumors compared to the TPA only-treated tumors (Fig. 6H).

## Discussion

ATRA is one of the most effective suppressors of tumor formation in the 2-stage skin carcinogenesis model [8,9]. ATRA and other retinoids have also been studied extensively for their potential as therapeutic and chemopreventive agents for a variety of cancers, including skin cancer [7,25-27]. However, in spite of the tremendous promise for retinoids in therapy and prevention of cancer, clinical results have often been disappointing due to hypervitaminosis A related side effects, leading to discontinuation of treatment. Our group has attempted to understand the chemopreventive mechanism of ATRA in order to more efficiently utilize these agents in the clinic, either alone or in combination with other drugs.

In this study we have used microarray technology to identify genes responsible for the tumor suppressive effect of ATRA. Specifically, we have used Affymetrix GeneChip^® ^Mouse Genome 430 2.0 arrays to probe mouse skin subjected to the 2-stage chemical carcinogenesis protocol for 3 weeks, with and without co-administration of ATRA. Among the TPA regulated genes identified, approximately half were oppositely regulated, or counter-regulated, by ATRA. We are calling these genes counter-regulated because their direction of expression (increase or decrease) in TPA + ATRA treated skin opposes that caused by TPA treatment alone.

Clustering the counter-regulated genes into functional categories has revealed that the MAP kinase signaling pathway was the single most disproportionately represented. This of course does not exclude the potential importance of other pathways that could mediate suppression of tumor promotion by ATRA. However, because elevated MAP kinase signaling has been implicated as one of the key events for epidermal carcinogenesis and TPA induced tumor formation [5,28-30], we chose to focus on this pathway initially. While the control of expression of genes in a particular signaling pathway and the control of signaling through that pathway are not identical processes, several previous microarray analyses have demonstrated a link between the two [31]. We also note that the KEGG pathway term 'MAPK signaling pathway' that is over-represented in our counter-regulated gene groups includes genes that are upregulated downstream of the B-Raf/Mek/Erk kinases (Table S1, additional file 2). We therefore considered these microarray results to be a suitable guide for deciding which pathway(s) to analyze in the context of this tumor model.

Since AP1 activity is stimulated by the MAP kinase pathway [32], this implicates suppression of this pathway and/or AP1 activity as the mechanism for the anti-tumor effect of ATRA [18]. A number of different mechanisms have been suggested, which include a RAR dependent exclusion of Erk and CREB-binding protein from a promoter containing AP1 binding sites [33], a restriction of nuclear entry of activated Erk1/2 [34], a repression of Erk1 gene expression at the level of transcription [35], an inhibition of Erk1/2 kinase activity [36], and inhibition of epidermal growth factor receptor (EGFR), a key upstream activator of MAP kinase signaling [37]. Further evidence of the importance of this pathway as a target for ATRA in epithelial carcinogenesis comes from a recent study that showed that ATRA could suppress proliferation of esophageal cancer cells through activation of retinoic acid receptor β2 (RAR-β2); and that this effect coincided with a suppression of EGFR/Erk/AP1 signaling and suppression of cyclooxygenase 2, a proinflammatory enzyme that is over-expressed in several cancer types [38]. In this study we show for the first time that in addition to blocking TPA regulation of expression of genes in the B-Raf/Mek/Erk pathway, ATRA also blocks TPA-induced signaling through the pathway at both early and late times during the 2-stage protocol.

The stress activated MAP kinase pathways (JNK and p38) have often been observed to counteract malignant transformation [39]. Consistent with those findings, we saw that unlike for B-Raf/Mek/Erk proteins, TPA treatment did not increase phosphorylation of either p38 or JNK. However ATRA treatment alone does enhance p38 phosphorylation at Thr180/Tyr182 (Fig. 5A, ATRA panels, and 5B, ATRA lane). This suggests that the tumor suppressive activity of ATRA potentially involves activation of p38 signaling. Further experiments are planned to explore this possibility.

Interestingly, the ATRA resistant tumors were histologically distinct from TPA induced papillomas, closely resembling advanced skin SCC (Fig. 3B). This is in agreement with previous findings by other investigators who showed that ATRA resistant tumors in the 2-stage model were at higher risk for conversion to carcinomas [40]. Because the conversion rate of papillomas to carcinomas is typically quite low, the numbers of mice used in our study were not sufficient to statistically determine whether TPA+ATRA papillomas had a higher conversion rate to carcinomas than TPA papillomas. The ATRA resistant tumors also expressed markedly lower levels of phospho-B-Raf, -Mek1/2, and -Erk1/2 than the TPA papillomas and TPA treated skin (data not shown), indicating that activation of this pathway is not required for survival of these tumors. We hypothesize that the ATRA resistance may be due to circumvention of the B-Raf/Mek/Erk pathway and that an alternate pro-proliferative pathway, such as the Stat3 pathway, is involved in the development of these tumors.

The mouse skin 2-stage model has been combined with DNA microarray technology by several investigators to more precisely define the gene expression changes related to skin carcinogenesis. Of particular relevance to our study is a report describing the gene expression profiles from C57BL/6 mouse skin exposed to a single treatment with TPA, using a cDNA microarray containing 5000 genes and a subtracted hybridization technique [41]. These investigators identified numerous previously unrecognized TPA-regulated genes, as well as genes shown to be up-regulated in advanced skin cancer. We note that several of these genes were identified in 3 week TPA treated skin in our study, in spite of the use of different mouse strains, microarray platforms and TPA treatment regimens. These include the TPA upregulated genes JunB, Saa3 (serum amyloid A3), Il4Rα (interleukin 4 receptor α), S100a9 (calgranulin B), Rrad (Ras-related associated with diabetes), Krt2-6a (keratin complex 2, basic, gene 6a), and others; as well as TPA downregulated genes such as Doc2γ (double C2, gamma), Aebp1 (AE binding protein 1) and others. In another study using the 2-stage model, investigators identified genes whose expression was altered in TPA treated skin, TPA-induced papillomas and SCCs, compared to control skin [42]. These investigators further determined by mRNA *in situ *hybridization that several of these genes were similarly altered in human skin SCC samples, indicating the relevance of the 2-stage model for understanding human skin cancer. Some of these genes, such as Lcn2 (lipcalin 2) and S100A8 (S100 calcium binding protein A8) were also upregulated in 3 week TPA treated skin (data not shown). Other studies have been conducted using microarrays to identify tumor associated genes in human cell lines and tissue. One group has directly compared genes differentially expressed between normal cultured keratinocytes and skin SCC cell lines, alongside a comparison of normal skin and skin SCC samples [43]. Although there was little overlap between the two systems, some of the tumor specific genes were found in both SCC cell lines and tissue. In another study using human samples, investigators compared the gene expression profiles between normal skin, psoriatic skin and skin SCC [44]. Some of the genes found to be commonly upregulated in psoriasis and skin SCC, such as ADAM23, Krt16 (cytokeratin 16) and IVL (involucrin), were also found to be upregulated in 3 week TPA treated skin (data not shown). Psoriasis is a benign hyperplastic disorder of the skin which bears some similarity to the hyperplastic state of the 3 week TPA treated skin used in our microarray study. However, there were also some genes that were only upregulated in skin SCC, such as EREG (epiregulin) and MMP13 (matrix metalloproteinase 13), that were also upregulated in 3 week TPA treated skin (data not shown). A cursory comparison of our data to the above studies identifies few TPA-ATRA counter-regulated genes, but mainly genes upregulated by TPA without alteration by ATRA. A more thorough comparison between these studies and our findings for TPA treated skin is planned. In the present study we have focused on genes and pathways that are counter-regulated by TPA and ATRA, rather than on the entire set of genes that are TPA regulated.

There is often an inverse correlation between the degree of squamous differentiation and malignancy in many cancers, including skin SCC. This observation has given rise to the concept of differentiation therapy, whereby the normal differentiative state of tumor cells can be restored by treatment with agents known to regulate differentiation such as ATRA [45]. We observed that tumors treated with Sorafenib, in addition to having suppressed Erk1/2 phosphorylation, have undergone extensive hyperkeratinization (Fig. 6A and 6B). This suggests that Sorafenib could potentially act as a tumor suppressor in skin SCC through induction of squamous differentiation and the accompanying exit from the cell cycle that results from blocking B-Raf/Mek/Erk signaling. Future experiments will explore the effects of Sorafenib in the 2-stage and UV induced skin carcinogenesis models, and will compare them directly with the effects of ATRA.

## Conclusion

We have shown that approximately half of the TPA regulated genes in mouse skin at an early pre-malignant stage in the 2-stage protocol are oppositely affected when ATRA is co-administered. These 'counter-regulated' genes provide the first expression signature for the chemopreventive effect of ATRA in a mouse skin cancer model. This is a potential source for novel targets for ATRA and other chemopreventive and therapeutic agents that can eventually be tested in the clinic. In addition, ATRA targets the B-Raf/Mek/Erk signaling pathway in the 2-stage model and this activity coincides with its chemopreventive action. This demonstrates the potential for targeting the B-Raf/Mek/Erk pathway for chemoprevention and therapy of skin SCC in humans. This is further supported by our finding that a pharmacological inhibitor of B-Raf results in the suppression of TPA-induced tumors.

## Methods

### 2-stage chemical carcinogenesis protocol

Young female outbred SENCAR mice were obtained from the National Cancer Institute (Frederick, MD) and were housed in a temperature- and humidity-controlled AAALAC facility with a 12 h light/dark cycle. All procedures were approved by the LSUHSC Institutional Animal Care and Use Committee in accordance with NIH guidelines. Mice were maintained on AIN-76A diet (Dyets, Bethlehem, PA), and allowed access to food and water *ad libitum*. Mice were treated as follows. For the microarray screening experiment, groups of 5 mice per treatment were shaved on the dorsal side and two days later initiated with 2.56 μg (10 nMol) 7,12-dimethylbenz [a]anthracine (DMBA) in 200 μl HPLC grade acetone, followed by twice weekly applications of 1 μg (1.62 nmol) TPA in 200 μl acetone, TPA plus 5 μg ATRA (16.67 nmol) in 200 μl acetone, ATRA alone, or acetone solvent only for 3 weeks. For the tumorigenesis and tissue analysis experiment, groups of mice were treated as above, except that the TPA treatment was carried out for 30 weeks. At the 3, 7 and 10 week time points, groups of 5 mice for each treatment group were sacrificed 6 hours after the last treatment, and the skin and tumors harvested for protein and tissue isolation. Tumor incidence (number of mice bearing tumors/total number of mice in treatment group) and the tumor multiplicity (average number of tumors per mouse) were recorded weekly for an additional set of mice (n = 5/group) until the 30 week time point. Tumor-bearing SENCAR mice that had been treated topically with TPA, twice weekly for 27 weeks, were treated with Sorafenib as follows. One set of mice were treated with the tosylate salt of Sorafenib (BAY 54-9085) by oral gavage according to [24], with one subgroup receiving 15 mg/kg and the other 30 mg/kg, twice weekly. Sorafenib was administered immediately following topical TPA treatment, which was continued throughout the experiment. The other set of mice were treated topically with Sorafenib, by direct application to the tumors, twice weekly. One subgroup received 0.3 μM Sorafenib in acetone solvent and the other received 0.6 μM. As for the gavage treatment, topical Sorafenib was administered immediately following TPA application. Tumor-bearing TPA treated mice were given topical acetone solvent alone, also immediately following TPA treatment, as controls. After three weeks of treatment with Sorafenib and TPA, skin and tumor tissue was isolated from all mice, 6 hours after the last set of treatments.

ATRA, DMBA, and TPA were obtained from Sigma (Sigma Chemical Co., St. Louis, MO). Sorafenib (BAY 43-9006) and it tosylate salt (BAY 54-9085) were generously supplied by Bayer Pharmaceutical Division (West Haven, CT).

### Immunohistochemical examination of mouse skin and epidermal thickness

Dorsal skin from mice in the each of the treatment groups was isolated and fixed in formalin and embedded in paraffin prior to sectioning. Paraffin sections of 4 μm were deparaffinized in xylene, 3 × 7 minutes, room temperature and rehydrated by stepwise washes in decreasing ethanol/H_2_O ratio (100% to 50%, followed by soaking in water). Sections were cut and stained with hematoxylin and eosin (H&E). For immunohistochemical staining, sections were incubated in Superblock (Pierce Biotechnology Inc., Rockford, Ill.) blocking reagent for 1 hour at room temperature to block non-specific antigen sites. After washing 3× in PBS, slides were incubated overnight at 4°C with phospho-specific antibodies against B-Raf (Ser 445), Mek1/2 (Ser 217/221), Erk1/2 (Thr202/Tyr204), SAPK/JNK (Thr183/Tyr185), p38 MAPK (Thr180/Tyr182) (Cell Signaling Technologies Inc.), and cytokeratin 10 (DAKO). Slides were photographed on a Nikon TE300 fluorescence microscope under oil immersion, at 600× magnification, with a CCD camera (Roper Scientific). Images were processed with IPLabs v3.55 software (Scanalytics Inc.). Epidermal thickness was measured using digital images of H&E stained skin sections, photographed at 400× magnification. Ten randomly selected fields were measured for each data point, using the rectangular marquee tool in Photoshop to determine the number of vertical pixels from the bottom of the basal cell layer to the beginning of, but not including, the stratum corneum. The length of each pixel was determined to be 0.169 μM, using a digital image of a hemocytometer grid, also photographed at 400×, for calibration.

### Skin RNA isolation

Total cell RNA was isolated by homogenization of snap frozen skin in TriReagent (Molecular Research Center Inc., Cincinnatti, OH), followed by standard organic extraction and precipitation and purification on RNeasy RNA purification columns (Qiagen Inc., Valencia, CA). Purity and yield were determined using an Agilent 2100 Bioanalyzer (Agilent Technologies).

### RNA labeling and GeneChip hybridization

Fluorescent probe synthesis and array hybridization was performed in the LSUHSC-S DNA Array Research Core Facility laboratory using established methods provided by Affymetrix (Affymetrix Inc., Santa Clara, CA). Briefly, 2 μg of purified total cell RNA, pooled from 3–5 mice in each treatment group, was reverse transcribed into cDNA, using a T7 promoter-(dT)24 primer. Following second strand synthesis, biotin-labeled cRNA was generated from the double stranded template using T7 polymerase. The quality of the cRNA probe was verified by running an aliquot on an agarose gel. Exactly 20 μg of the labeled cRNA was hybridized to the Affymetrix GeneChip^® ^Mouse Genome 430 2.0 Array for 16 hours at 45°C in 300 μl of pre-mixed hybridization solution containing labeled hybridization control prokaryotic genes (bioB, bioC, bioD, and cre). Several duplicate spots for each control gene are present on the chip. Chips were washed in the GeneChip Fluidics Station automatic washer and scanned on the GeneArray^® ^fluorometric scanner.

### DNA microarray analysis and visualization

Data extraction and examination of the raw intensity data was conducted within the LSUHSC-S DNA Array Core Facility using MicroArray Suite 5.0 (Affymetrix Inc.) and the freeware software package dChip (from Dr. Wing Wong's laboratory, ). Gene expression data sets were further analyzed using GeneSifter (VizX Labs, Seattle, WA) and NetAffx (Affymetrix Inc.) microarray analysis software packages in addition to proprietary algorithms and tools developed at LSU-Shreveport. Differential expression and subsequent visualization of genes counter-regulated by TPA and ATRA was performed based on the fold change between three pairs: the signal intensity value of the TPA-treated skin (TPA) with skin treated with acetone solvent alone (control), TPA+ATRA treated skin with TPA, and between TPA+ATRA and control. Scatterplots were drawn for each of these three sets of comparisons (Fig. S1, additional file 1). Each scatterplot represents a gene as a single dot, with unchanged genes appearing on the diagonal where the log-transformed x and y values are equal or very similar. Linear boundaries parallel to the x = y diagonal line represent the 2-fold and 4-fold borderlines. We considered only genes for which the p value ≤ 0.05 for signals within a probe set for a given gene, and for which there was a present call for either control, TPA treated skin, or both. We first identified the genes regulated by TPA treatment relative to untreated controls and divided these into 2 clusters, C1 (increased in expression ≥ 1.5-fold by TPA treatment) and C2 (decreased in expression ≥ 1.5-fold by TPA treatment). The C1 group was further divided into three subclusters; C1sA are genes suppressed by ≥ 1.5-fold upon TPA+ATRA co-administration compared to TPA alone; C1B are genes that remain relatively unchanged upon co-administration of TPA+ATRA relative to TPA alone, and C1C are genes that are further induced by ≥ 1.5-fold by TPA+ATRA relative to TPA alone. The C2 cluster was also divided into three subclusters. C2A are genes induced by ≥ 1.5-fold upon TPA+ATRA co-administration compared to TPA alone; C2B are genes that remain relatively unchanged upon co-administration of TPA+ATRA relative to TPA alone, and C2C are genes that are further suppressed by ≥ 1.5-fold by TPA+ATRA relative to TPA alone. The expression patterns for each of these groups are shown in the graphs in Fig. 2, alongside the corresponding heatmap. For this figure, we normalized each gene to a mean of 0 and a standard deviation of 1, and utilized sorting based on the similarity of each gene's three measurements (control, TPA and TPA+ATRA). To obtain the heatmap representation, each gene expression value is represented graphically by coloring a cell in the table on the basis of the measured fluorescence ratio. Unchanged genes are colored black, while up-regulated genes are shown in red and down-regulated genes in green.

The complete microarray datasets for control, TPA-, ATRA-, and TPA+ATRA-treated samples have been entered into the LSU Health Science Center MicroArray Database, which is MIAME compliant and administered by the Laboratory for Advanced Bioinformatics at the Department of Computer Sciences at LSU-Shreveport.

### Western blotting

Mouse epidermis was scraped from isolated dorsal skin on ice and homogenized with a Polytron for three 10 sec bursts in RIPA lysis buffer (150 mM NaCl, 50 mM Tris-HCl pH 7.5, 1 mM EDTA, 1% NP-40, 1 mM PMSF, 1 mM Na-orthovanadate) supplemented with 40 μl Complete protease inhibitor cocktail (Roche, Indianapolis, IN) according to manufacturer-provided instructions. Extracted protein was quantified using the Bio-Rad Protein Assay kit (Hercules, CA). Proteins were separated by SDS acrylamide gel electrophoresis (12%) and transferred to nitrocellulose membranes (Schleicher & Schuell, Dassel, Germany). Blots were blocked with 5% BSA for 1 h at room temperature, followed by incubation overnight with antibodies against phosphorylated forms of B-Raf (Ser 445), Mek1/2 (Ser 217/221), Erk1/2 (Thr202/Tyr204), SAPK/JNK (Thr183/Tyr185), and p38 MAPK (Thr180/Tyr182); and total B-Raf, c-Raf, Mek1/2, Erk1/2, p39 and SAPK/JNK; and human β-actin (all from Cell Signaling Technology, except for B-Raf and β-actin from Santa Cruz Biotechnology). Blots were washed with TBS/0.1% Tween 20 and incubated with horseradish peroxidase-conjugated secondary antibody for 1 h at room temperature, followed by 3 additional washes with TBS/0.1% Tween 20. Chemiluminescence detection was performed according to the manufacturer's instructions (Pierce, Rockford, Ill.), followed by exposure to X-ray film.

## Competing interests

The authors declare that they have no competing interests.

## Authors' contributions

SBC carried out immunohistochemistry, skin RNA and protein isolation, Western blotting, microarray data analysis, and contributed to the draft of the manuscript. WY contributed to the performance of immunohistochemistry, Western blotting, and the draft of the manuscript. ZY contributed to the immunohistochemistry and microscopy, as well as to the harvesting of tumors and skin. JNG performed the 2-stage carcinogenesis experiments and assisted with immunohistochemistry and tissue harvesting. AM assisted with 2-stage experiments, immunohistochemistry, and maintenance of the mice. HEK participated in experimental design and writing of the manuscript. ML contributed to the design of Sorafenib experiments and interpretation of data. MT and UC contributed to the design of the microarray experiment and microarray data analysis. JLC conceived of the study, coordinated the study, contributed to the immunohistochemistry, microscopy, tissue harvesting, microarray data analysis and contributed to the draft of the manuscript.

## Supplementary Material

Additional file 1 **Scatterplots indicate the relationship between the six gene subclusters; C1A (blue), C1B (red), C1C (yellow), C2A (violet), C2B (green), and C2C (pink)**. Diagrams showing a comparison of gene expression between the same control mouse skin and TPA treated skin shown in Fig. 2 (A). A comparison of gene expression between TPA treated skin with skin treated with TPA + ATRA (B). A comparison of gene expression between control mouse skin and skin treated with TPA + ATRA (C). x and y axes indicate log-transformed expression values. Lines parallel to (*x *= *y*) diagonal represent 2-fold and 4-fold borderlines. The parallel gaps in A and B are the result of excluding genes which show ≥ 1.5-fold difference in expression between TPA treated skin and skin treated with TPA + ATRA, but are not found in the C1B and C2B subclusters. When comparing control skin with TPA + ATRA skin, the majority of counter-regulated genes lie close the x = y diagonal, indicating that these genes, while induced or suppressed in expression by TPA treatment alone, are expressed at similar levels in control skin and skin treated with TPA + ATRA (Fig. 3C, see C1A and C2A subclusters). Click here for file

Additional file 2 **Transducers of TPA effects that are oppositely regulated by TPA and ATRA**. The table shows average raw signals from the A430 2.0 GeneChips for selected genes from the C1A and C2A subclusters in Fig. 2, arranged by functional categories. Gene ontology was determined using the GeneSifter microarray analysis software package. Green shading indicates lower expression relative to red shading. Genes found in more than one category are included. The signaling pathways shown in the table are those with the highest Z-scores for either the C1A, C2A or both clusters, for that. Z-score is a measure of the significance of over-representation for a particular gene list within an ontology group [21]. Only genes for which a statistically significant score (P ≤ 0.01 within a probe set) was obtained for at least one treatment group are shown. Click here for file

Additional file 3 **Previously identified TPA regulated genes that are oppositely regulated by TPA and ATRA**. The table shows average raw signals for selected genes represented as in Table 1. Click here for file

Additional file 4 **Expression of C-Raf and phospho-C-Raf in SENCAR mouse skin**. The figure shows an Western blot for which epidermal lysates were prepared as in Fig. 4B from SENCAR mouse skin treated with Acetone (Con), TPA, TPA+ATRA, or ATRA alone for 7 weeks. Total protein (15 μg per well) from pooled samples (n = 5) was run on 10% SDS-PAGE and probed with antibodies for total C-Raf, phospho-Ser338 C-Raf, and β-actin. Click here for file
